# Case Report: A case study of geriatric acute cholecystitis complicated by coinfection with *Shewanella putrefaciens* and *Enterococcus faecium*

**DOI:** 10.3389/fmed.2026.1792551

**Published:** 2026-03-05

**Authors:** Jiawei Liu, Yu Zhan, Yingmiao Zhang, Tian Feng, Hui Wang, Zhongxin Lu

**Affiliations:** 1 Department of Medical Laboratory, The Central Hospital of Wuhan, Tongji Medical College, Huazhong University of Science and Technology, Wuhan, China; 2 Key Laboratory for Molecular Diagnosis of Hubei Province, The Central Hospital of Wuhan, Tongji Medical College, Huazhong University of Science and Technology, Wuhan, China

**Keywords:** acute cholecystitis, co-infection, *Enterococcus faecium*, procalcitonin, *Shewanella putrefaciens*

## Abstract

**Background:**

Acute cholecystitis is a common abdominal condition mainly caused by enteric Gram-negative bacilli and *Enterococcus* species. Advances in microbial detection have highlighted infections by rare pathogens like *Shewanella putrefaciens* (*S. putrefaciens*), an opportunistic bacterium from aquatic environments affecting mainly immunocompromised or comorbid patients. Its clinical features, antibiotic resistance, and treatment remain unclear.

**Case presentation:**

This article presents a case study of an 87-year-old female patient with a medical history of gallstones and previous endoscopic retrograde cholangiopancreatography (ERCP), who was admitted to the hospital due to “low back and leg pain.” On November 14, 2023, she developed acute cholecystitis. Initial treatment consisted of cefoperazone/sulbactam and ciprofloxacin. Ultrasound-guided percutaneous transhepatic gallbladder drainage (PTGBD) was performed, revealing purulent bile that tested positive for *S. putrefaciens* and *Enterococcus faecium* (*E. faecium*). Based on susceptibility testing, the antibiotic regimen was adjusted to cefoperazone/sulbactam and vancomycin, which was administered until November 24, 2023. The patient’s condition subsequently improved, and she was discharged from the hospital.

**Conclusion:**

We documented the inaugural case of an elderly patient presenting with acute cholecystitis co-infected with *S. putrefaciens* and *E. faecium*. This case underscores the importance of integrating source control via PTGBD with targeted antimicrobial therapy guided by drug susceptibility testing, highlighting their synergistic role in effective management. Furthermore, the monitoring of procalcitonin (PCT) levels offers valuable support for clinical decision-making.

## Background

Acute cholecystitis represents a prevalent abdominal emergency, primarily attributed to enteric Gram-negative bacilli and *Enterococcus* species. In recent years, advancements in microbial detection technologies and an enhanced clinical understanding have led to increased reports of atypical or opportunistic pathogens in biliary tract infections, among which *S. putrefaciens* is notable. This bacterium is ubiquitously found in aquatic environments and generally functions as an opportunistic pathogen, predominantly affecting immunocompromised individuals or those with multiple underlying comorbidities. Infections of the biliary tract by *S. putrefaciens* are rare, with limited consensus on their symptoms, antibiotic resistance, and treatment (1, 2). A review up to 2011 found only 10 cases of biliary tract infections among 256 *Shewanella* infections, highlighting their rarity (3). A 2023 review analyzing literature up to March 2022 found 87 cases of confirmed or suspected *S. putrefaciens* infection, with only 4 involving the biliary tract and 1 involving an infected pancreatic pseudocyst (1). This highlights the rarity of biliary tract infections caused by *S. putrefaciens*. These cases often involve polymicrobial infections in patients with existing hepatobiliary issues (4). Notably, a co-infection of *S. putrefaciens* with *E. faecium* causing acute cholecystitis has yet to be clearly documented.

In this report, we present the first documented case of acute cholecystitis resulting from a co-infection with *S. putrefaciens* and *E. faecium*. The patient, an elderly female with multiple chronic comorbidities, was admitted to the hospital for treatment of acute cholecystitis that developed during her hospitalization. Bile culture and antimicrobial susceptibility testing revealed a mixed infection with *S. putrefaciens* and *E. faecium*. The initial empirical anti-infective therapy was complicated by discrepancies between the drug susceptibility results and the antibiotics recommended by clinical guidelines, highlighting the need for personalized treatment strategies in the context of quinolone resistance and intrinsic enterococcal resistance. Furthermore, serial PCT measurement served as a valuable adjunct tool to assess therapeutic response, and its trend informed the decision-making regarding antibiotic duration alongside comprehensive clinical evaluation. By meticulously documenting the diagnostic and therapeutic process, as well as the microbiological characteristics, this case aims to provide clinical insights for the management of biliary tract infections involving similarly uncommon pathogens.

## Case presentation

**Figure 1:**
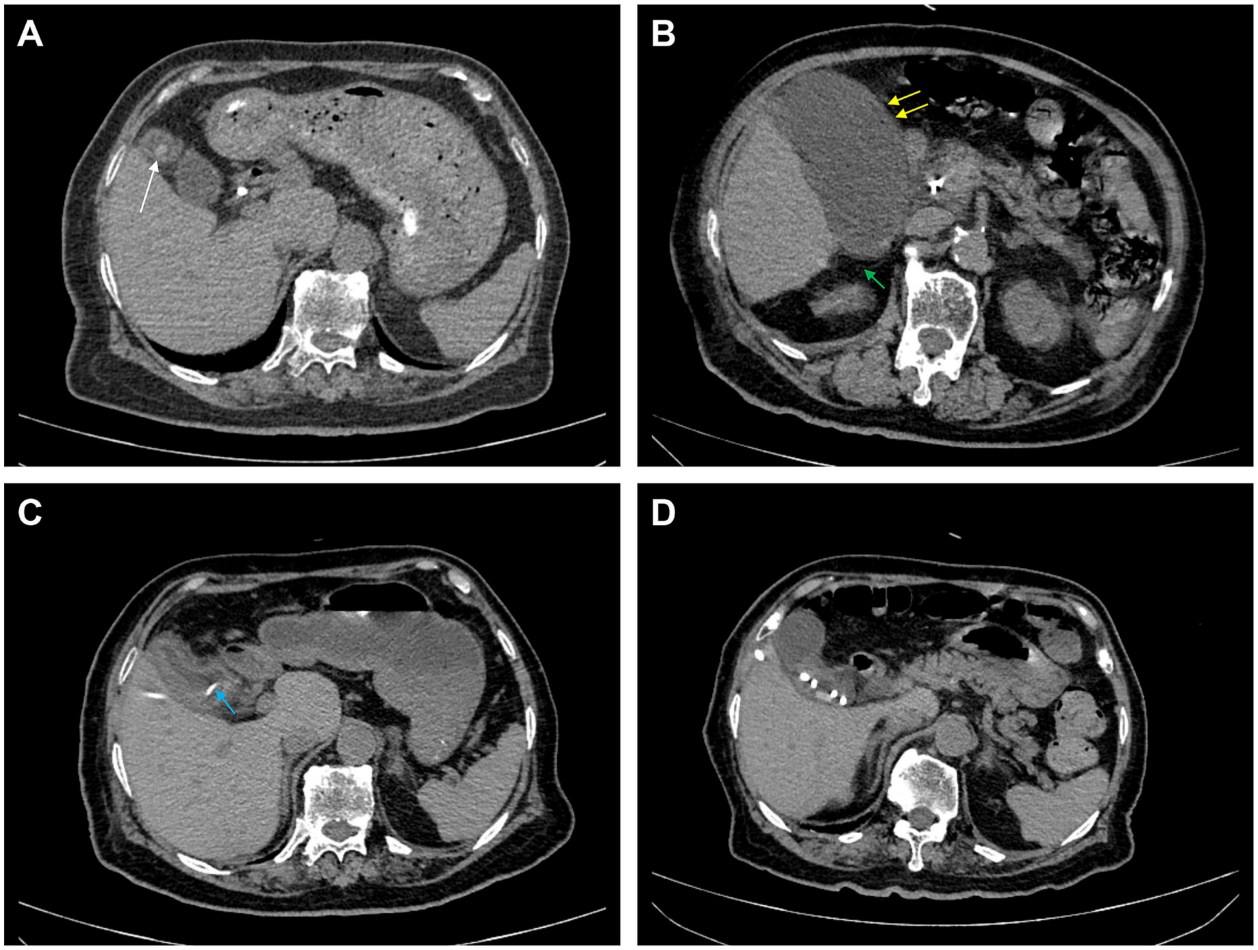
Abdominal CT scan of the patient post admission. **(A)** Baseline (3 months before admission): CT shows gallstones (white arrow) with a normal gallbladder and no inflammation. **(B)** Acute phase (4 days post admission): CT reveals a swollen gallbladder with wall thickening (yellow arrowhead) and significant surrounding stranding (green arrow). **(C)** Post-drainage (9 days post admission): CT after PTGBD shows a decompressed gallbladder with a drainage catheter (blue arrow). **(D)** One-month follow-up: Gallbladder remains contracted and thickened.

An 87-year-old female patient was admitted on November 10, 2023, due to lumbar and leg pain, with a recent history of a high-fat diet. A previous abdominal Computed Tomography (CT) scan had indicated the presence of cholecystitis and cholecystolithiasis, without clear evidence of common bile duct dilation (Figure 1A). Two years earlier, she had developed acute suppurative cholangitis due to the impaction of a common bile duct stone at the duodenal papilla, which was alleviated by ERCP with nasobiliary drainage. On the fourth day of hospitalization (November 14, 2023), the patient experienced an acute exacerbation of cholecystitis, characterized by a low-grade fever (37.8 °C), abdominal muscle rigidity, tenderness, and rebound pain in the right upper quadrant. A follow-up CT scan revealed significant enlargement of the gallbladder, diffuse wall thickening, and increased pericholecystic effusion compared to previous studies (Figure 1B). Laboratory findings demonstrated an elevated white blood cell count (13.36 × 10^9^/L) with 85.10% neutrophils, and a C-reactive protein (CRP) level of 21.80 mg/L. Alanine aminotransferase, alkaline phosphatase, bilirubin, and lipase levels remained within normal reference ranges.

The patient was admitted to the intensive care unit due to advanced age and multiple comorbidities, including coronary atherosclerotic heart disease, chronic heart failure, stage 3 hypertension, and stage 3 chronic kidney disease. Given that secondary cholecystitis infections are predominantly attributed to Gram-negative enteric bacilli and enterococci, an intravenous regimen of cefoperazone/sulbactam (3 g/12 h) in combination with ciprofloxacin (0.4 g/12 h) was initiated on November 14, 2023. On the same day, ultrasound-guided PTGBD was performed, yielding dark green purulent bile with abundant biliary sediment; the drainage fluid was subsequently sent for microbiological culture. The bile specimen was cultured on blood and MacConkey agar, incubated at 35 °C with 5% CO₂ for 24 h. Dominant colonies were identified via matrix-assisted laser desorption/ionization time-of-flight mass spectrometry (MALDI-TOF MS), and antimicrobial susceptibility was tested using the Kirby-Bauer method. Following the PTGBD, there was a notable reduction in the white blood cell count, which decreased from 17.32 on November 16, 2023, to 7.98 on November 18, 2023. MALDI-TOF MS identified the presence of *S. putrefaciens* and *E. faecium*. The absence of bacterial growth in both anaerobic and aerobic blood cultures obtained during the febrile phase suggests that the infection was localized exclusively to the biliary tract. The results of antimicrobial susceptibility testing, conducted on November 18, 2023, are detailed in Tables 1, 2.

**Table 1:** Results of antimicrobial susceptibility testing for *S. putrefaciens.*

Antibiotic	K-B (mm)	Interpretation
Gentamicin	18	S
Ciprofloxacin	6	R
Meropenem	34	S
Ceftazidime	33	S
Cefoperazone/Sulbactam	32	S
Aztreonam	30	S
Amikacin	22	S
Imipenem	28	S
Piperacillin	21	S
Cefepime	31	S
Levofloxacin	13	R
Piperacillin/Tazobactam	32	S

K-B, Kirby-Bauer method; S, sensitive; I, intermediate; R, resistant.

**Table 2:** Results of antimicrobial susceptibility testing for *E. faecium.*

Antibiotic	K-B (mm)	Interpretation
Tetracycline	10	R
Teicoplanin	18	S
Levofloxacin	6	R
Vancomycin	18	S
Penicillin	6	R
Ampicillin	6	R
Linezolid	24	S
Ciprofloxacin	6	R

K-B, Kirby-Bauer method; S, sensitive; I, intermediate; R, resistant.

On November 19, 2023, the patient’s vital signs stabilized, and the abdominal pain resolved. Physical examination indicated a soft, non-tender abdomen without rebound tenderness. A follow-up CT scan demonstrated a reduction in gallbladder volume, although wall thickening, edema, and blurred margins persisted (Figure 1C). Nodular hyperdense shadows within the gallbladder lumen remained unchanged, and the drainage tube was appropriately positioned. Laboratory tests showed a normalized white blood cell count (8.05 × 10^9^/L) and neutrophil percentage (69.40%), but elevated levels of CRP (60.56 mg/L), lipase (120 U/L), and PCT (4.94 ng/mL) persisted. Based on antimicrobial susceptibility testing, the antibiotic regimen was adjusted to cefoperazone/sulbactam (3 g/12 h intravenously) in combination with vancomycin (0.5 g/12 h intravenously). On November 24, 2023, the patient was in stable condition with normal vital signs. Abdominal pain had lessened, and bowel and urinary functions were normal. Heart and lungs showed no issues, and there was no jaundice. The abdomen was soft and flat, with normal bowel sounds and no tenderness. The legs were not swollen. Anti-infection treatment was maintained until that date, and lab results are shown in Table 3.

**Table 3:** Laboratory results of the patient.

Parameter	Values									Normal range
	Nov. 14	Nov. 15	Nov. 16	Nov. 17	Nov. 18	Nov. 19	Nov. 20	Nov. 23	Nov. 26	
WBC (×10^9/L)	13.36	17.58	17.32	11.16	7.98	8.05	10.30	7.54	8.38	3.5 ~ 9.5
N (%)	85.10	89.20	88.10	84.20	78.80	69.40	71.90	62.20	69.50	40 ~ 75
TBIL (μmol/L)	13.9	–	14.1	–	–	13.4	13.3	9.0	6.8	5.1 ~ 19
ALT (U/L)	10.0	–	7.7	–	–	9.6	17.6	25.6	32.9	7 ~ 40
AST (U/L)	34.5	–	23.9	–	–	19.7	31.5	30.2	42.7	13 ~ 35
GGT (U/L)	86.0	–	68.2	–	–	67.8	89.2	103.0	99.6	7 ~ 45
ALP (U/L)	114.0	–	–	–	–	75.0	–	–	–	40 ~ 150
Lipase (U/L)	31	–	–	–	–	120	130	141	145	13 ~ 60
CREA (μmol/L)	128.4	129.2	133.9	113.1	104.0	109.4	102.4	91.4	116.2	41 ~ 81
CRP (mg/L)	21.80	–	–	–	–	60.56	39.11	–	–	0 ~ 6
INR	0.95	1.15	1.21	1.15	1.14	1.13	–	1.39	1.24	0.7 ~ 1.3
PCT (ng/mL)	–	–	–	17.17	–	4.94	2.48	–	<0.1	0 ~ 0.1

WBC, white blood cell; N, neutrophil; TBIL, total bilirubin; ALT, alanine aminotransferase; AST, aspartate aminotransferase; GGT, gamma-glutamyl transferase; ALP, alkaline phosphatase; CREA, Creatinine; CRP, C-reactive protein; INR, international normalized ratio; PCT, procalcitonin. “–” indicates that the assay was not done.

**Figure 2:**
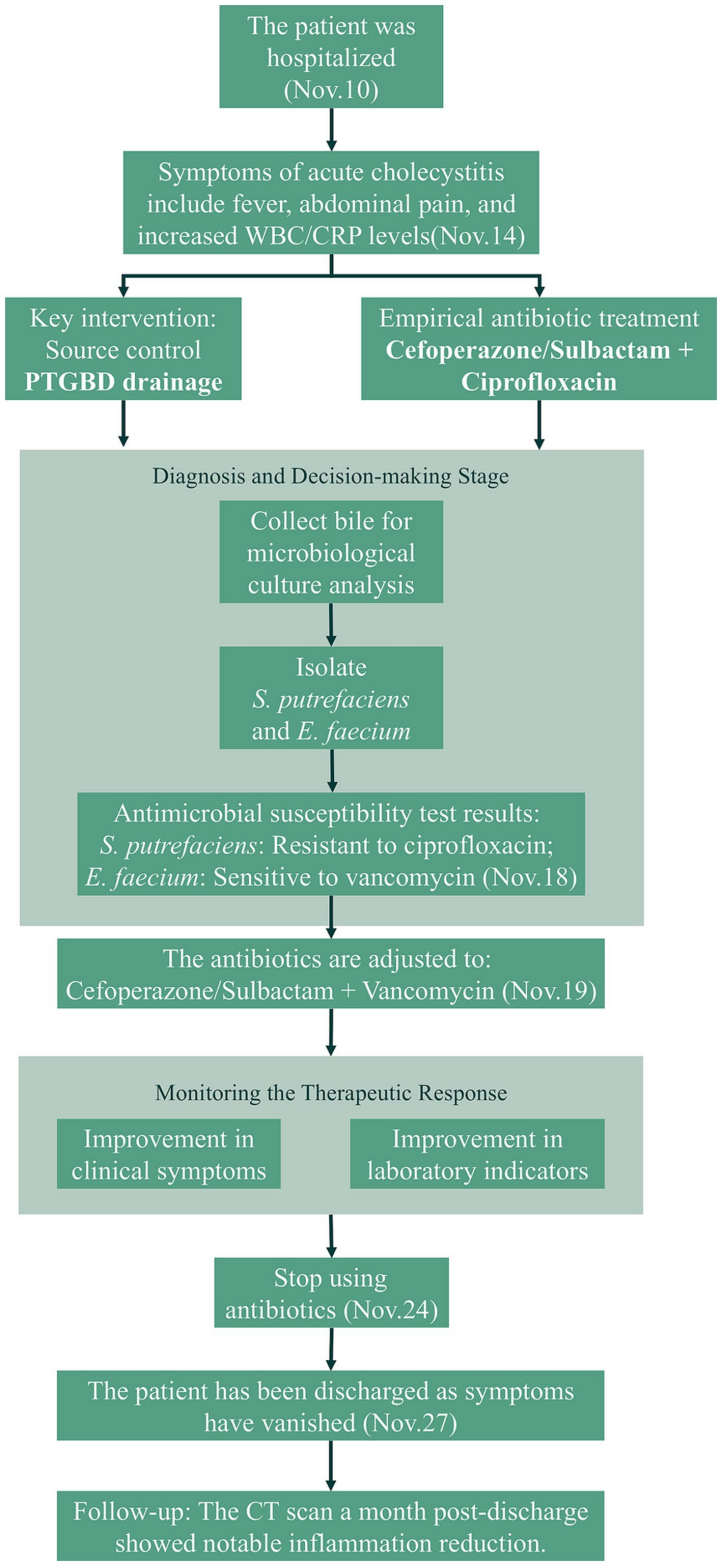
Clinical management flowchart of the case presentation.

On November 27, 2023, the patient’s vital signs were stable, with a complete resolution of abdominal pain and tenderness. Laboratory parameters indicated a normal white blood cell count (8.38 × 10^9^/L) and PCT level (<0.1 ng/mL). The patient was discharged after successful treatment with antibiotics and drainage therapy. A follow-up CT scan conducted 1 month later demonstrated significant resolution of gallbladder and pericholecystic inflammation (Figure 1D). The patient’s clinical management flowchart is illustrated in Figure 2.

## Discussion

*S. putrefaciens*, a Gram-negative bacillus naturally found in aquatic environments, is an opportunistic pathogen in humans. Infections are commonly associated with seawater exposure and typically manifest as skin/soft tissue or intra-abdominal infections, which may progress to bacteremia (1). Notably, biliary tract involvement is exceedingly rare. Table 4 highlights the rarity of *S. putrefaciens*-associated cholecystitis or biliary infections in the literature. Four reported cases involved patients with biliary stones and polymicrobial infections, including *Escherichia coli* and *Klebsiella pneumoniae* (4). Another review emphasizes the rarity and notes that hepatobiliary disease is a major risk factor for severe *Shewanella* infections, such as bacteremia with high mortality (3). Other sporadic reports also link biliary infections or bacteremia to cholelithiasis or cirrhosis, with a common factor being underlying hepatobiliary pathology and frequent polymicrobial infections (5, 6). In alignment with this pattern, our elderly female patient presented with cholecystolithiasis and a polymicrobial infection. Despite her lack of direct exposure to seawater, her immunocompromised condition, resulting from multiple comorbidities, is acknowledged as a recognized risk factor (6–8). It is noteworthy that *Shewanella* species often colonize bile as part of a polymicrobial community (9), and cholestasis induced by biliary obstruction provides an environment conducive to bacterial proliferation.

**Table 4:** Summary of reported cases of biliary tract/cholecystitis caused by *S. putrefaciens*.

Reference	Case number	Patient Age/Sex/Details	Major underlying disease(s)	Type of infection	Treatment	Co-infection pathogens
Chen et al. (1997) (4)	1	83, M (Pt 11)	Biliary stones, Prostate cancer	Biliary tract infection with liver abscess	CFZ + GEN ± MTZ; later to CPZ + CLN	*E. coli*
Chen et al. (1997) (4)	1	52, M (Pt 12)	Biliary stones	Biliary tract infection with liver abscess	CFZ + GEN + MTZ	*K. pneumoniae*
Chen et al. (1997) (4)	1	71, M (Pt 13)	Biliary stones	Biliary tract infection	CFZ + GEN	*E. coli, B. fragilis, Enterococcus* spp.
Chen et al. (1997) (4)	1	81, M (Pt 14)	Biliary stones	Biliary tract infection	CFZ + GEN + MTZ	*K. pneumoniae*
Vignier et al. (2013) (3)	2	Details N/R	Details N/R	Cholecystitis	Details N/R	Details N/R
Goyal et al. (2011) (5)	1	45, M	Cholelithiasis	Acute cholecystitis	Antibiotics N/S; Laparoscopic cholecystectomy	Co-isolation N/S
Tsai et al. (2008) (6)	1	74, M	Bile duct stones, Liver cirrhosis	Bacteremia, Necrotizing fasciitis (primary source possibly biliary)	TZP, later to CRO	Co-isolation N/S
Present case	1	87, F	Cholecystolithiasis, CAD, CHF, HTN grade 3, CKD stage 3	Acute cholecystitis	CPZ/SBT + CIP; later to CPZ/SBT + VAN	*E. faecium*

Antibiotics: CFZ (Cefazolin), GEN (Gentamicin), MTZ (Metronidazole), CPZ (Cefoperazone), CLN (Clindamycin), TZP (Piperacillin/Tazobactam), CRO (Ceftriaxone), CPZ/SBT (Cefoperazone/Sulbactam), CIP (Ciprofloxacin), VAN (Vancomycin).

Pathogens: *E. coli* (*Escherichia coli*), *K. pneumoniae* (*Klebsiella pneumoniae*), *B. fragilis* (*Bacteroides fragilis*).

Clinical: M (Male), F (Female), Pt (Patient), N/R (Not reported), N/S (Not specified), CAD (Coronary artery disease), CHF (Chronic heart failure), HTN (Hypertension), CKD (Chronic kidney disease).

Timely source control was crucial for successful management in this case. The patient, with a history of gallstones and previous ERCP, faced a risk of biliary obstruction. Performing PTGBD aligned with the Tokyo Guidelines 2018 (TG18), which advise gallbladder drainage for acute cholecystitis when surgery is not an option. This procedure relieved the obstruction, drained purulent bile, and reduced the white blood cell count (10). TG18 emphasizes biliary drainage as essential for treating acute biliary infections with obstruction or suppuration, primarily to lower biliary pressure and endotoxin exposure (11). Here, antibiotics played a supportive role.

Within this therapeutic framework, and following the establishment of definitive source control via PTGBD, empirical antibiotic therapy was initiated in accordance with the TG18 to cover the predominant pathogens in acute cholecystitis, namely Enterobacteriaceae and Enterococcus. However, antimicrobial susceptibility testing indicated that *S. putrefaciens* was resistant to ciprofloxacin but susceptible to cefoperazone/sulbactam. Concurrently, the co-existing *E. faecium* demonstrated resistance to quinolones while remaining susceptible to vancomycin. Additionally, due to the intrinsic resistance of *E. faecium* to cephalosporins, cefoperazone/sulbactam was ineffective against this pathogen. TG18 also recommends vancomycin as the preferred empirical agent for *E. faecium* (12). Consequently, an adjusted therapeutic regimen combining cefoperazone/sulbactam with vancomycin was implemented to effectively target both pathogens. This targeted adjustment was intended to eradicate the specific residual pathogens after source control had been achieved, thereby consolidating the therapeutic effect rather than serving as the primary curative intervention. Although no formal guidelines exist for the treatment of *Shewanella* infections, and significant strain-dependent variations in resistance patterns are observed, existing literature suggests a general susceptibility of *Shewanella* to third- and fourth-generation cephalosporins and carbapenems (3, 13).

Research has shown that plasmid-mediated quinolone resistance genes, such as QnrA, naturally originate from *Shewanella* species found in aquatic environments (14). Within this genus, the chromosomal presence of innate Qnr homologs demonstrates variable expression levels, influenced by regulatory sequences and gene integrity. This variability provides a plausible explanation for the heterogeneous susceptibility to quinolones observed in clinical isolates (14). *E. faecium* is naturally resistant to cephalosporins because of the low-affinity penicillin-binding protein Pbp5 and signal transduction systems like CroRS and IreK (15–19). Enterococci often resists quinolones through gyrA and parC mutations, efflux pump activation, and Qnr-like proteins (20–23).

Another critical consideration is that the bile culture in this case was conducted several days following the commencement of empirical treatment with cefoperazone/sulbactam and ciprofloxacin. The application of these broad-spectrum antibiotics may have imposed a selective pressure on the biliary microbiome, potentially suppressing or eliminating strains susceptible to antibiotics, such as certain *Enterobacteriaceae*. As a result, bacteria that were either resistant or intrinsically resistant to the initial therapy-specifically, *S. putrefaciens*, which is resistant to ciprofloxacin, and *E. faecium*, which is intrinsically resistant to cephalosporins-emerged as the predominantly cultivated organisms (14). Consequently, the culture results are likely indicative of the microbial profile under antibiotic selective pressure and may not accurately represent the full spectrum of pathogens present at the onset of infection. Therefore, initiating timely empirical antibiotic therapy is crucial for acute biliary infections, but obtaining bile or relevant specimens for culture early on is also important. This helps confirm pathogen eradication and guide treatment adjustments if clinical response is poor or infection recurs.

PCT is a superior tool for monitoring therapeutic response and guiding antibiotic duration compared to traditional markers, due to its specificity for bacterial infections and rapid kinetics. Large randomized controlled trials (RCTs) and meta-analyses support this approach. In critically ill patients, PCT-guided strategies, such as stopping antibiotics when PCT decreases by ≥80% or is ≤0.5 μg/L, safely reduced antibiotic duration from 7 to 5 days without increasing mortality (24). A meta-analysis of 26 RCTs showed PCT guidance cuts antibiotic use by 2.4 days and may lower 30-day mortality (25). An earlier RCT found PCT guidance reduced antibiotic use in lower respiratory infections by 51% without affecting outcomes (26).

To effectively use this evidence in clinical practice, it’s crucial to understand the biomarker’s nature and limitations. PCT mainly indicates the host’s systemic inflammatory response, not pathogen clearance or the success of interventions like antibiotics or source control. Its reduction results from the combined effects of these interventions and the immune response, making it hard to attribute changes to a single factor (27). Therefore, PCT should not replace a thorough clinical evaluation. Research shows that even when a PCT threshold is met, clinicians rely on their overall assessment of the patient’s stability, highlighting PCT as a supplementary tool rather than a definitive guide (24, 27). The decision to stop antibiotics should be based on PCT level trends, confirmed infection control, and resolved symptoms. In this case, decreasing PCT levels matched clinical improvement, highlighting PCT’s value in assessing treatment response. Notably, PCT levels dropped significantly (from 17.17 ng/mL on November 17 to 4.94 ng/mL on November 19) before vancomycin was added, indicating that source control (PTGBD) was the main factor in clinical improvement. The antibiotic adjustment aimed to eliminate remaining pathogens but cannot solely be credited with a “curative” effect.

Despite the presence of confirmed biliary obstruction, the patient’s serum levels of alanine aminotransferase (ALT), alkaline phosphatase (ALP), and total bilirubin (TBil) remained within normal limits throughout the clinical course. This finding strongly indicates that PTGBD was effective in alleviating the biliary obstruction as a timely source control intervention, thereby preventing the onset of obstructive jaundice and secondary liver injury. Furthermore, although serum lipase levels were transiently elevated to 120 U/L on November 19, the patient did not exhibit any clinical symptoms or radiological evidence suggestive of acute pancreatitis. Considering that cholecystitis itself may cause mild pancreatic inflammation, this transient elevation is more likely attributable to an infection-associated nonspecific response rather than an independent diagnosis of pancreatitis (28).

## Patient perspective

As an 87-year-old with multiple health issues, including heart disease and heart failure, I was hospitalized for lower back and leg pain. During my stay, I experienced an acute cholecystitis attack, causing severe pain and anxiety. The medical team quickly performed PTGBD and administered antibiotics, relieving my symptoms. My treatment was later adjusted to a targeted antibiotic regimen based on culture results. Following a course of treatment, my condition showed improvement, leading to my discharge from the hospital. The attending physician clearly explained my condition and treatment changes to me and my family, ensuring effective communication throughout.

## Data Availability

The original contributions presented in the study are included in the article/supplementary material, further inquiries can be directed to the corresponding author.
